# A randomized controlled trial on the effect of focal thermal therapy at acupressure points treating osteoarthritis of the knee

**DOI:** 10.1186/s13018-021-02398-2

**Published:** 2021-04-27

**Authors:** Kevin Ki-Wai Ho, Anthony Wai-Leung Kwok, Wai-Wang Chau, S. M. Xia, Y. L. Wang, Jack Chun-Yiu Cheng

**Affiliations:** 1 Department of Orthopaedics and Traumatology, Chinese University of Hong Kong, Hong Kong, SAR China; 2 School of Medical & Health Sciences, Tung Wah College, Hong Kong, SAR China

**Keywords:** Knee osteoarthritis, Acupressure, Randomized controlled trial

## Abstract

**Background:**

Osteoarthritis of the knee is a common degenerative joint disorder in our ageing population. A combination of thermal therapy with a self-management exercise have shown a positive effect in the management of osteoarthritis of the knee. This study aimed to compare the effectiveness of topical heat pack versus focal application of heat therapy at the acupressure points in the treatment of osteoarthritis of the knee.

**Methods:**

A randomized controlled trial was conducted in 76 patients with osteoarthritis of the knee, diagnosed by an experienced orthopedic surgeon. Following inclusion and exclusion selection, patients were randomly allocated to group 1 (Heat pack) or group 2 (Thermal gun). All patients received 30 min of treatment in each session, twice a week for 4 weeks. They also received an education program and taught home knee exercises. Outcome measurements were the visual analog scale (VAS) for pain intensity, muscle power, knee ROM, WOMAC and SF-12v2.

**Results:**

In the Thermal gun group, function and total scores (WOMAC) and Physical Composite Scale (SF-12v2) were significantly improved after 8 sessions. Quadriceps strength was significantly improved after 8 weeks (from 4.42 to 4.63; *p* = 0.02). In the Heat pack group, flexion was significantly improved after 8 sessions (*p* = 0.02). Mean VAS scores after Heat pack treatment was consistently better (lower) than mean VAS scores after Thermal gun treatment.

**Conclusion:**

The combination of focal thermal therapy at acupressure points is a viable conservative treatment in osteoarthritis of the knee. The pressure at the acupressure points has a synergistic benefit than topical thermal therapy alone.

**Trial registration:**

ClinicalTrials.gov, NCT04735029

Date of registration: February 2, 2021 (Retrospectively registered)

## Background

Osteoarthritis (OA) of the knee is a common degenerative joint disorder in our ageing population. It is estimated that nearly 70% of the population at age 70 years or older have clinical symptoms or radiological evidence of osteoarthritis of the knee [1]. Females have a higher prevalence of the disease than males, of which nearly 15% of middle-aged and elderly patients in both developed and developing countries are affected, resulting in a main social and economic burden in the healthcare service [2, 3]. With the increasing number of both the obesity and ageing population, more people were more likely to develop OA of the knees [3].

Symptoms of knee pain with reduced joint mobility may result in decreasing physical activities and sedentary lifestyle [4]. All these physical and psychological symptoms frequently lead to depression and poor quality of life [5, 6].

Patients with end-stage osteoarthritis of the knee would normally resort to a total knee replacement (TKR). However, there are many combinations of pharmacologic and non-pharmacologic therapies before surgery. These include physical therapy, nonsteroidal anti-inflammatory medication and activity modification [7, 8]. However, these may cause unpleasant side effect and gastrointestinal disturbance [9]. Therefore, topical therapy is introduced to provide a better option to reduce any systemic side effects [10].

A combination of hot and cold therapy with self-management exercise have shown a positive effect in the management of osteoarthritis of the knee [11]. Similar techniques can be found in Tai Chi exercise, moxibustion, herbal therapy and acupressure [12, 13].

This study aims to compare the effectiveness of topical heat pack versus focal application of heat therapy to acupressure in the treatment of osteoarthritis of the knee.

## Methods

This study was conducted in compliance with the Declaration of Helsinki and was approved by The Joint Chinese University of Hong Kong—New Territories East Cluster Clinical Research Ethics Committee (CREC Ref. No: 2016.555-T). In the completion of this study, it was audited by the Joint CUHK-NTEC Clinical Research Management Office (CRMO) on 29 September 2018. The study was retrospectively registered in February 2021 with ClinicalTrials.gov (Registration number: NCT04735029).

This randomized controlled study was conducted at the Prince of Wales Hospital, Hong Kong from 2017 to 2018. Consecutive patients visiting the Orthopaedics Specialist Outpatient Clinic with symptomatic end-stage OA of the knee on queue for scheduling TKR procedures were entitled to participate in the study. All patients enrolled into this study have end-stage osteoarthritis of the knee with Kellgren-Lawrence grade IV on X-rays. The inclusion criteria were (1) male or female patients aged 40 years old or older, (2) clinical and radiological diagnosis of osteoarthritis of the knee based on the OA Knee clinical guidelines and radiological evidence [14, 15], (3) normal skin sensation to heat, cold, pins and pricks (i.e. passed the required Skin Sensation Test), (4) not participating in any other clinical trial at time of this study, (5) be able to complete the whole trial period, and (6) no cognitive dysfunction and was able to sign the consent form. The exclusion criteria were (1) stages 1 & 2 OA of the knee, (2) received bilateral knee arthroplasty before this study, (3) polyarthritis affecting more than both knee, (4) active skin lesion, (5) pregnant or breastfeeding women, (6) received acupuncture and/or moxibustion within 1 month of the study and (7) unable to comply with the study protocol. If a patient presented with bilateral OA knee symptoms, only the most symptomatic knee was treated with the study protocol for evaluation. Analgesic and NSAIDs were not strictly prohibited as many patients with end-stage OA of the knee did suffer from severe knee pain and required analgesic for pain relief. The randomization process, in turn, eliminated the bias caused by NSAID intake.

### Sample size calculation

The estimated study sample size is 76. This calculation was based upon a previous pilot study assumption of the required confident interval is 95%, type I error is 5%, and the statistic power is 80%. Hence, 38 patients were assigned to either group 1 (Heat pack) or group 2 (Thermal gun).

### Randomization

The allocation concealment was carried out using the sealed envelope system.

### Intervention

**Fig. 1:**
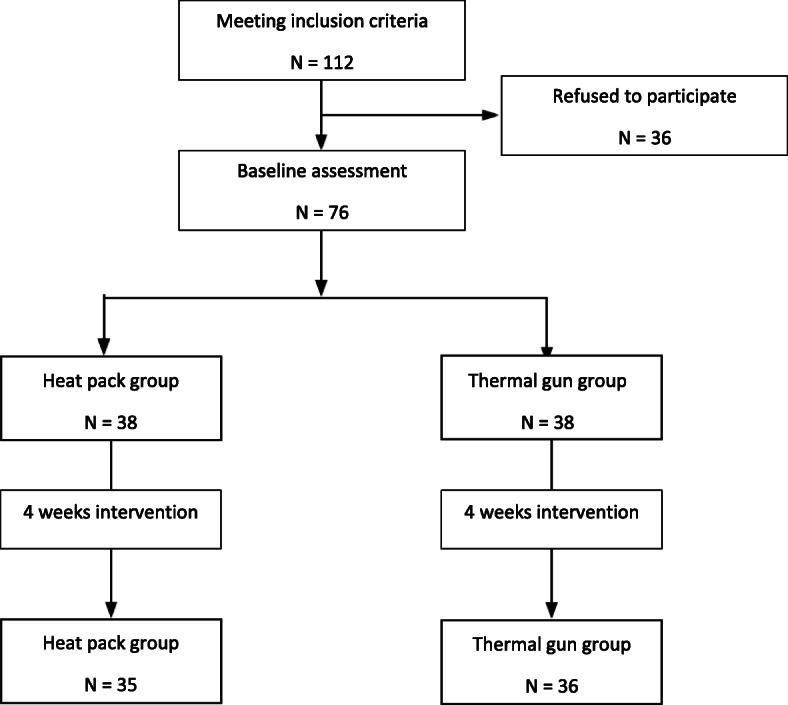
The summary of the study recruitment

Subjects were randomized to receive either topical heat pack treatment (Group 1: Heat pack group) as in the traditional thermal therapy or focal thermal gun treatment (Group 2: Thermal gun group) at some selected acupressure points (Fig. 1). The thermal gun was originally developed and made by the Hong Kong Productivity Council and passed the safety standards requirements under the instructions from the Inventor and received Utility Patent Number ZL201310022959.0 dated 27 January 2016 from the State Scientific and Technological Commission, Peoples’ Republic of China (Certificate Number: 1935113). The principle of the thermal gun application on acupressure points was the use of a heat-generating ceramic head to apply a dual effect called “heat-and-light-pressure” on the acupressure points. The heat could relieve knee pain and stiffness, improve blood circulation and relax the tight muscle [16]. This philosophy of “acupressure” was similar to “acupuncture” without the invasiveness of needle puncture in the traditional acupuncture techniques [17, 18]. All subjects were tested positive from the Skin Sensitivity Test to heat and cold prior to this study and were taught a home program of quadriceps muscle strengthening and hamstring stretch exercises. A standardized exercise instruction sheet, with diagram illustration, was also handed to each subject. A trained physiotherapist was involved to train quadriceps and hamstring muscle strengths. A standardized home instruction sheet with diagram illustration was also delivered.

In the heat pack group, a hydrocollator heat pack was used to simulate traditional thermal therapy. The heat pack was wrapped with six layers of terry towels to bring the temperature down to around 43 °C. The temperature of the heat pack would drop over time, but the treatment time was the same in both groups at 30 min and the layers of towels minimized heat loss.

**Fig. 2:**
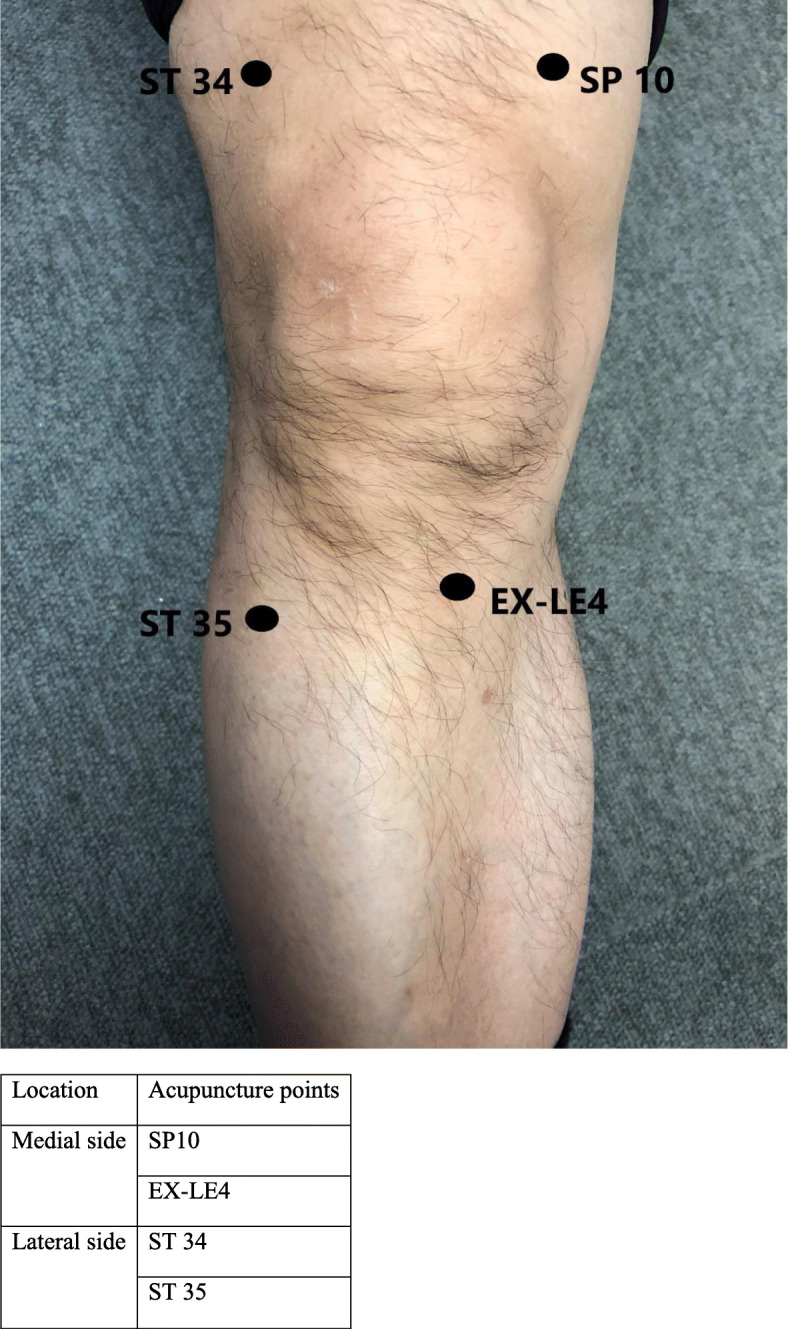
Acupressure points around the knee. On the medial side, acupuncture points are SP10 and EX-LE4. On the lateral side, acupuncture points are ST34 and ST35

In the thermal gun group, the thermal gun was used as a focal thermal treatment on the acupressure points. Subjects in the thermal gun group were given a localized thermal treatment from the thermal gun on specified acupressure points. The thermal gun generated a temperature of 43°C at the ceramic tip of the device. The specific acupressure points (Fig. 2) were SP10, ST 34, ST35 and EX-LE4, and the most tender spot/Trigger point, selected based on the previous study on the positive effect of focal thermal therapy. Subjects were taught how to identify the locations of the selected acupressure points and the amount of pressure to be applied onto each acupressure point using an educational and feedback method by a physiotherapist. The thermal gun treatment was done by self-administration. Each of the 5 acupressure points were required to be pressed and ran for 5 min, and the total duration of treatment was 30 min.

### Outcome assessment

**Fig. 3:**
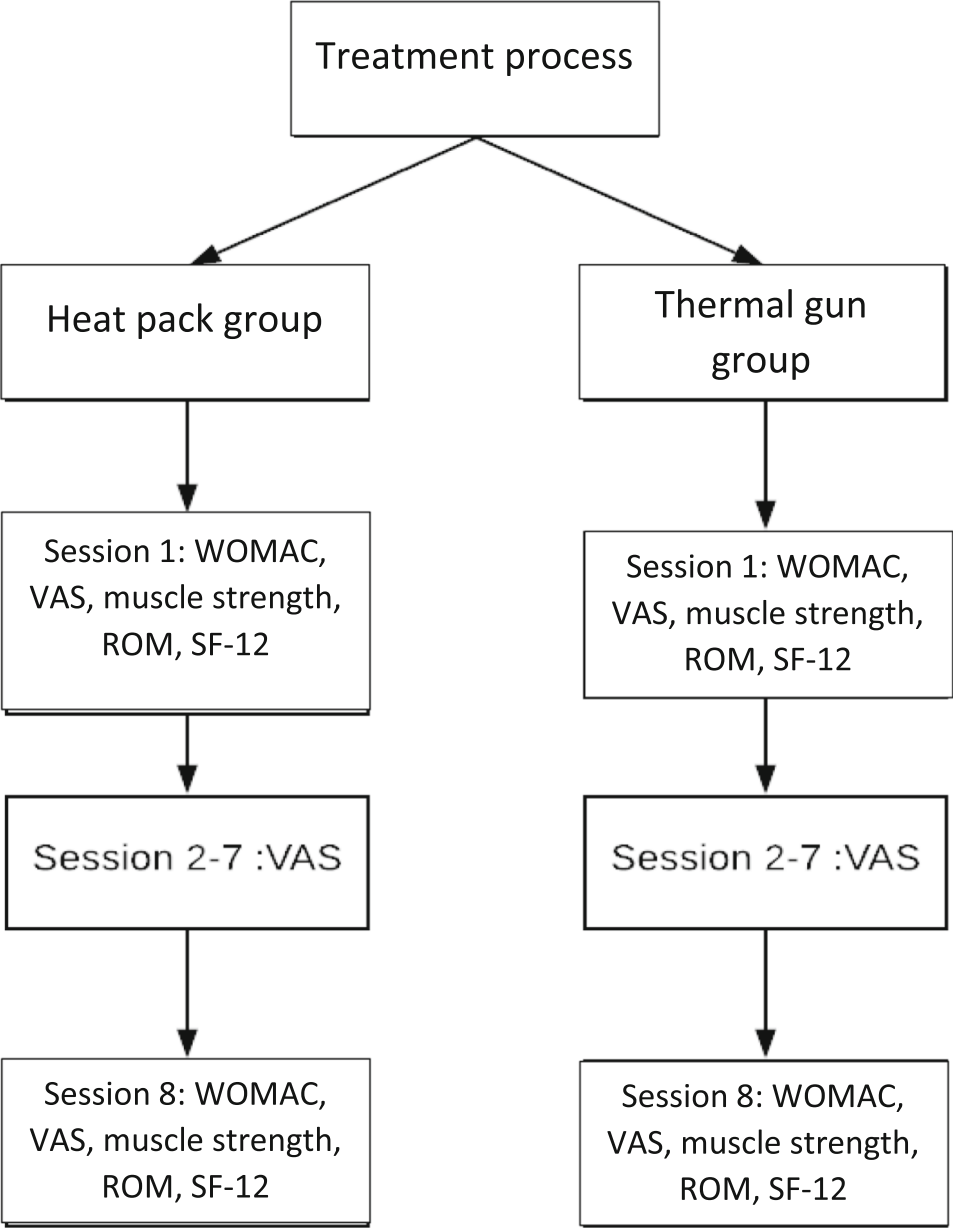
The workflow of the study

All measurements were recorded by the same investigator to maintain accuracy and consistency. Patients’ demographic data and the outcomes assessment were recorded before and after the intervention. The following outcome assessments were recorded: (1) visual analogue scale (VAS) for pain, (2) knee range of movement in the supine position using a goniometer, (3) quadriceps and hamstring muscle strength were measured according to the Oxford Scale of Muscle Strength, (4) functional assessment with the validated Chinese version of the Western Ontario and McMasters Universities (WOMAC) and Short Form-12 version 2 (SF-12v2) [19] (Fig. 3).

### Data analysis

Demographic characteristics were described by mean and standard deviation for numeric variables and *N* (%) for categorical variables where appropriate. Independent *T* tests were used to compare the differences between baseline and after 8 sessions of intervention within the same group, as well as between-group comparisons. In-depth comparative analysis of VAS scores taken after each of the 8 treatment sessions were compared based on (1) between-group using Student’s *T* test and (2) within the 8 sessions using ANOVA. This was followed by *post hoc* Bonferroni multiple adjustments to look for significances under multiple comparisons. All statistical analyses were carried out using IBM SPSS version 24 (Armonk, NY: IBM Corp). Statistical significance was set at *p* < 0.05.

## Results

One hundred and twelve subjects were recruited and attended the screening assessment of the study. Thirty-six subjects did not meet the inclusion criteria due to various reasons. The remaining 76 subjects were randomly assigned equally to the Thermal gun group (*N* = 38) as the intervention group and the Heat pack group (*N* = 38) as the control. A summary of the subjects’ demographic was shown in Table 1. Subjects treated with the thermal gun suffered from significantly longer pain duration than patients treated with a heat pack (mean years = 10.00 vs. 7.15, *p* = 0.04); otherwise, no difference was noticed in all other basic characteristics. All the patients have symptoms that affected their activity of daily living.

**Table 1:** Basic characteristics of subjects in the two groups

Variables	Categories		Thermal gun (*N* = 38)	Heat pack (*N* = 38)	*p* value
Age			66.58 ± 7.38	68.00 ± 6.16	0.37
Gender	Male		8 (21%)	13 (34%)	0.31
	Female		30 (79%)	25 (66%)	
Symptomatic side	Right		24 (63%)	22 (58%)	0.82
	Left		14 (37%)	16 (42%)	
Duration (year)	Mean ± SD		10.00 ± 6.20	7.15 ± 4.44	0.04
Occupation	Wholesale and retail trades, restaurants and hotels		8 (21%)	12 (31%)	0.25
	Community, social and personal service		16 (42%)	14 (37%)	
	Transport and related service, storage and communication		5 (13%)	4 (11%)	
	Manufacturing		0	2 (5%)	
	Construction		0	2 (5%)	
	Civilian service		2 (5%)	0	
	Stay at home		7 (19%)	4 (11%)	
Capable activities	Kneeling	Cannot	29 (76%)	30 (79%)	1.00
		Can	9 (24%)	8 (21%)	
	Squatting	Cannot	20 (53%)	23 (61%)	0.64
		Can	18 (47%)	15 (39%)	
	Carry weight	Cannot	24 (63%)	21 (55%)	0.64
		Can	14 (37%)	17 (45%)	
	Running	Cannot	34 (89%)	36 (95%)	1.00
		Can	4 (11%)	2 (5%)	
	Vigorous knee movement	Cannot	37 (97%)	37 (97%)	1.00
		Can	1 (3%)	1 (3%)	
	Prolonged standing	Cannot	27 (71%)	22 (58%)	0.34
		Can	11 (29%)	16 (42%)	
	Walking	Cannot	28 (74%)	26 (68%)	1.00
		Can	10 (26%)	12 (32%)	

* The differences of intergroup were calculated by the paired *T* test.

### WOMAC

**Fig. 4:**
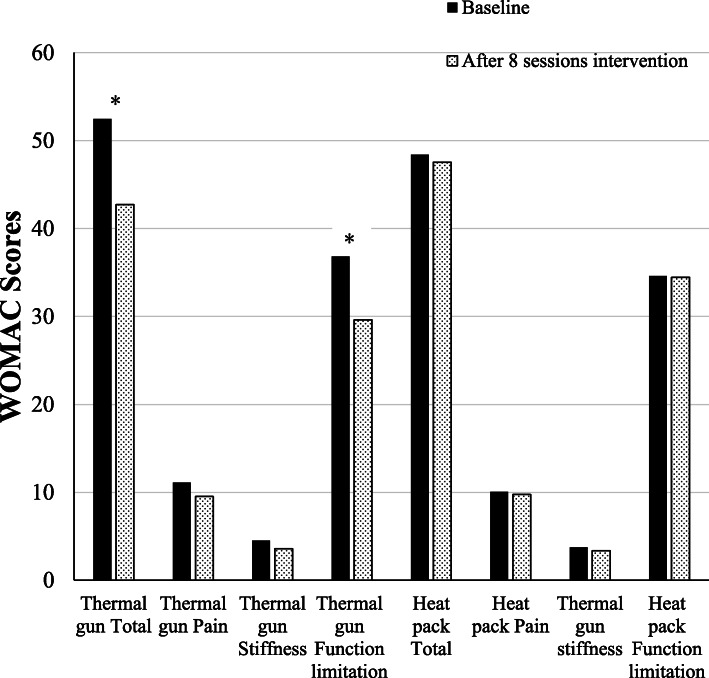
The intergroup change of WOMAC

In the Thermal gun group, WOMAC function limitation and total scores were significantly improved after 8 sessions of interventions (both *p* = 0.02) (Table 2, Fig. 4). Pain and stiffness scores were also improved although statistical significances had not been reached. No significance was found in any comparison in the Heat pack group. For between-group comparison, no statistically significant difference was found between the Thermal gun and Heat pack groups (Table 3).

**Table 2:** Comparisons of WOMAC, mean VAS, SF-12v2, knee ROM, and muscle strength between groups pre and post 8 sessions’ intervention

Variables	Group		Baseline (Mean ± SD)	After 8 sessions’ intervention (Mean ± SD)	*p* value
WOMAC	Thermal gun	Total	52.45 ± 17.76	42.71 ± 16.14	0.02^*^
		Pain	11.11 ± 3.62	9.52 ± 3.50	0.07
		Stiffness	4.50 ± 2.06	3.58 ± 1.80	0.06
		Function limitation	36.84 ± 13.42	29.61 ± 11.95	0.02^*^
	Heat pack	Total	48.39 ± 16.06	47.55 ± 13.74	0.84
		Pain	10.05 ± 3.34	9.75 ± 2.97	0.74
		Stiffness	3.74 ± 2.04	3.35 ± 1.95	0.49
		Function limitation	34.61 ± 11.73	34.45 ± 10.87	0.96
Mean VAS	Thermal gun		4.51 ± 2.39	4.12 ± 2.05	0.48
	Heat pack		4.27 ± 2.15	3.85 ± 1.80	0.46
SF-12v2	Thermal gun	PCS	29.07 ± 6.36	32.27 ± 5.28	0.04^*^
		MCS	36.25 ± 9.21	38.55 ± 7.76	0.31
	Heat pack	PCS	30.20 ± 8.65	30.38 ± 4.76	0.92
		MCS	35.99 ± 10.56	36.52 ± 7.60	0.86
Knee ROM	Thermal gun	Extension	14.61 ± 5.82	11.27 ± 4.85	0.01^*^
		Flexion	111.72 ± 14.62	113.50 ± 12.52	0.59
	Heat pack	Extension	11.03 ± 2.80	9.90 ± 1.75	0.07
		Flexion	110.05 ± 14.58	119.50 ± 11.01	0.02^*^
Muscle strength	Thermal gun	Quadriceps	4.42 ± 0.36	4.63 ± 0.39	0.02^*^
		Hamstring	4.66 ± 0.40	4.81 ± 0.31	0.09
	Heat pack	Quadriceps	4.67 ± 0.37	4.68 ± 0.41	0.97
		Hamstring	4.88 ± 0.22	4.88 ± 0.28	0.92

* *p* < 0.05

**Table 3:** Comparisons of WOMAC, mean VAS, SF-12v2, knee ROM, and muscle strength between “Thermal gun” and “Heat pack” groups

Variables	Group		Thermal gun	Heat pack	*p* value
WOMAC	Baseline (Mean ± SD)	Total	52.47 ± 17.58	48.39 ± 16.06	0.30
		Pain	11.11 ± 3.62	10.05 ± 3.34	0.19
		Stiffness	4.50 ± 2.06	3.74 ± 2.04	0.11
		Function limitation	36.84 ± 13.42	34.61 ± 11.73	0.44
	After 8 sessions’ intervention (Mean ± SD)	Total	42.71 ± 16.14	47.55 ± 13.74	0.27
		Pain	9.52 ± 3.50	9.75 ± 2.97	0.81
		Stiffness	3.58 ± 1.80	3.35 ± 1.95	0.67
		Function limitation	29.61 ± 11.95	34.45 ± 10.87	0.15
Mean VAS	Baseline		4.51 ± 2.39	4.27 ± 2.15	0.65
	After 8 sessions’ intervention		4.12 ± 2.05	3.85 ± 1.80	0.63
SF-12v2	Baseline	PCS	29.07 ± 6.36	30.20 ± 8.65	0.54
		MCS	36.25 ± 9.21	35.99 ± 10.56	0.91
	After 8 sessions’ intervention	PCS	32.27 ± 5.28	30.38 ± 4.76	0.25
		MCS	38.55 ± 7.76	36.52 ± 7.60	0.41
Knee ROM	Baseline	Extension	14.61± 5.82	11.03 ± 2.80	< 0.01^*^
		Flexion	111.72 ± 14.62	110.05 ± 14.58	0.62
	After 8 sessions’ intervention	Extension	11.27 ± 4.85	9.90 ± 1.75	0.16
		Flexion	113.50 ± 12.52	119.50 ± 11.01	0.09
Muscle strength	Baseline	Quadriceps	4.42 ± 0.36	4.67 ± 0.37	< 0.01^*^
		Hamstring	4.66 ± 0.40	4.88 ± 0.22	< 0.01^*^
	After 8 sessions’ intervention	Quadriceps	4.63 ± 0.39	4.68 ± 0.41	0.69
		Hamstring	4.81 ± 0.31	4.88 ± 0.28	0.42

* *p* < 0.05

### SF-12v2

The Physical Composite Scale (PCS) was significantly improved after applying thermal gun for 8 weeks (Mean PCS: 29.07 vs. 32.27, *p* = 0.04), while no significant difference was found in the Heat pack group (Table 2). No difference was found in all between-group comparisons (Table 3).

### Knee ROM

Flexion was significantly improved after 8 sessions of heat pack treatment (110.05 vs. 119.50, *p* = 0.02) (Table 2). The significant difference in knee extension at baseline was not found after the intervention (Table 3).

### Muscle strength

Quadriceps strength was significantly improved after the 8-week thermal gun treatment (increased from 4.42 to 4.63; *p* = 0.02) (Table 2). Both quadriceps and hamstring strengths on either left or right side became statistically insignificant after either treatment, despite finding significant differences before treatment (Table 3).

### Changes in VAS along the 8 sessions

**Fig. 5:**
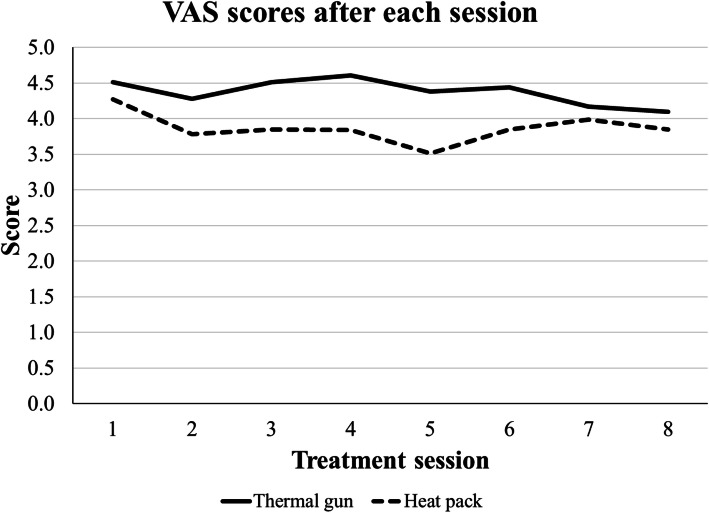
Longitudinal VAS scores obtained after each session from patients treated by either thermal gun or heat pack

VAS scores in every session were compared in two different ways: between treatments per session (Table 4), and across the 8 timepoints per treatment (Table 5). Mean VAS scores after heat pack treatment was consistently better (lower) than mean VAS scores after thermal gun treatment (Fig. 5), although statistical significance was nearly reached at Session 5 (3.51 (Heat pack) vs. 4.38 (Thermal gun), *p* = 0.09). No statistical significance was found in the longitudinal changes in both groups.

**Table 4:** Mean VAS scores in comparing between Thermal gun and Heat pack treatments at each session

Sessions	Thermal gun	Heat pack	*p* value
1	4.51 ± 2.39	4.27 ± 2.15	0.65
2	4.28 ± 2.10	3.78 ± 1.68	0.26
3	4.51 ± 2.14	3.85 ± 1.52	0.16
4	4.61 ± 1.91	3.84 ± 1.68	0.11
5	4.38 ± 2.02	3.51 ± 1.40	0.09
6	4.44 ± 1.97	3.85 ± 1.73	0.27
7	4.17 ± 2.09	3.99 ± 1.95	0.75
8	4.12 ± 2.05	3.85 ± 1.80	0.63

**Table 5:** Mean VAS scores in comparing among sessions within treatment

Treatment	Session								Overall *p* value
	1	2	3	4	5	6	7	8	
Thermal gun	4.51	4.28	4.51	4.61	4.38	4.44	4.17	4.12	0.98
Heat pack	4.27	3.78	3.85	3.84	3.51	3.85	3.99	3.85	0.89

### Adverse effect

No patient reported any physical discomfort or adverse effect during the study period in both groups.

### Dropout

Five subjects (2 from the Thermal gun group and 3 from the Heat pack group) failed to attend all the 8 sessions due to either personal or work reasons. The dropout rate was 6.6%.

### Full completion of the clinical trial

Thirty-five subjects (92.1%) in the Heat pack group and 36 subjects (94.7%) in the Thermal gun group completed all the 8 treatment sessions in 4 weeks.

## Discussion

This study evaluated the effects of combining the acupressure points and thermal therapy in the treatment of OA of the knees. Thermal gun therapy improved the physical functioning of the OA of the knees and the subject’s health-related quality of life. Heat pack treatment significantly improved knee flexion ROM and made improvement in knee extension ROM, though not significant when comparing with the Thermal gun group. Quadriceps were much improved in subjects who received thermal gun treatment.

Pain relief after thermal gun therapy are constantly better than in subjects after heat pack therapy. The pain level difference is at its highest after the fifth session. A systematic review provided a reliable evidence that acupressure was effective in relieving pain [20]. In the Thermal gun group, the combined effects of the acupressure and the topical heat provide more benefits than solely on the heat therapy from the heat pack. A further study with larger sample sizes in each group is recommended to prove this speculation. The significant improvements would possibly not be limited to the fifth session and the third and fourth sessions as well.

Thermal gun therapy improved general and physical functioning of the joints and patient’s health-related quality of life. In SF-12v2, improvement could only be found in the physical score of the Thermal gun group. Thermal gun also improved quadriceps and hamstring strengths significantly. There was no significant influence on mental health. The findings in this study were consistent with the previous study which showed beneficial effects on the physical health of women with dysmenorrhea after acupressure therapy, but not to mental health [21]. Yildirim et al. did a heat therapy trial and showed similar findings. Heat therapy was more sensitive to physical change instead of mental health. However, the trial from Yildirim did not show any change to patients’ quality of life after heat therapy [22]. The different result from Yildirim may be related to the period of treatment and heat therapy device. Localized thermal therapy may improve the quality of life through a higher pain relief effect.

Heat pack treatment significantly improved knee flexion ROM and also made improvement in knee extension ROM. The use of thermal gun significantly improved quadriceps strength over time. The differences in muscle strength found between the Thermal gun group and the Heat pack group vanished after the intervention. This may be due to the acupressure points located near the insertion of the quadriceps tendon. Acupressure with circular movement is like a massage that can relax the tight and tired muscles. Acupressure application on these points may relax the quadriceps muscle and relieve pain, resulting in increasing ROM of the knee. The home stretching exercise can increase the flexibility and range of motion [23]. The combination of increased ROM and pain relief helps to improve their physical function [24].

In this cohort, subjects received 8 treatment sessions in 4 weeks. Li et al. used self-administered acupressure over 8 weeks, resulting in a big reduction of pain and improvement in physical function for older adults with osteoarthritis of the knee [25]. This clinical trial showed similar results, but with a shorter timepoint as compared with their study. It seems that the actual duration of treatment may not be a critical factor in managing patients’ symptoms, but rather, the education and patient self-awareness [26]. Managing patients’ symptoms are multi-factorial, but education and self-empowerment are the best ways to treat chronic pain due to degenerative joints. Nevertheless, this study helped to understand this complementary therapy and provided an evidence-based alternate treatment in osteoarthritis of the knee.

There are some limitations in this clinical trial. All questionnaires were self-administered and had a strong element of subjectivity. The treatment cycle of 4 weeks might be shorter than in previous studies. That could be the major reason for not reaching statistical significance going through the 8 sessions (4 weeks). Managing patients’ symptoms are multi-factorial, but education and self-empowerment are the best ways to treat these patients. The short follow-up period limits the data generalizability on long-term benefit for this therapy. Finally, this was an open study that might induce expectations from both the researchers and the subjects. For further study, it is necessary to have a larger sample size and a longer follow-up period to truly assess the benefit of this treatment. Nevertheless, this study helped to understand this complementary therapy and provided an evidence-based alternate treatment in osteoarthritis of the knee.

## Conclusions

The combination of focal thermal therapy at acupressure points is a viable conservative treatment in OA of the knees. The pressure at the acupressure points has a synergistic benefit than topical thermal therapy alone. The thermal gun is more portable, self-temperature regulatory and convenient to apply than the topical thermal heat pack.

## Data Availability

The datasets used and/or analyzed during the current study are available from the corresponding author upon request.

## Abbreviations

OA: Osteoarthritis
TKR: Total knee replacement
VAS: Visual analogue scale
WOMAC: Western Ontario and McMasters Universities
SF-12v2: Short Form-12 version 2
ROM: Range of motion
